# Spatial Analysis of a Cat-Borne Disease Reveals That Soil pH and Clay Content Are Risk Factors for Sarcocystosis in Sheep

**DOI:** 10.3389/fvets.2019.00127

**Published:** 2019-04-24

**Authors:** Patrick L. Taggart, Mark A. Stevenson, Simon M. Firestone, Milton M. McAllister, Charles G. B. Caraguel

**Affiliations:** ^1^School of Animal and Veterinary Sciences, The University of Adelaide, Roseworthy, SA, Australia; ^2^Asia-Pacific Centre for Animal Health, Faculty of Veterinary and Agricultural Sciences, Melbourne Veterinary School, The University of Melbourne, Parkville, VIC, Australia

**Keywords:** *Sarcocystis*, *Toxoplasma*, point pattern, soil, pH, acidity, risk factor, feral cat

## Abstract

Cat-borne parasites and their associated diseases have substantial impacts on human, livestock, and wildlife health worldwide. Despite this, large and detailed datasets that allow researchers to study broad-scale trends in the ecology of cat-borne diseases are either difficult to obtain or non-existent. One condition that is easily detected at slaughter is macroscopic sarcocystosis, a cat-borne parasitosis of sheep (*Ovis aries*). We conducted a cross-sectional study to describe the geographic distribution of sarcocystosis in sheep throughout South Australia and investigate ecosystem characteristics associated with the presence of disease. Data were obtained from two slaughterhouses which processed 3,865,608 sheep from 4,204 farms across 385,468 km^2^ of South Australia's land mass for the period 2007–2017. A Poisson point process model was developed to quantify environmental characteristics associated with higher densities of sarcocystosis-positive farms. Sarcocystosis was highly clustered on a large island off of the Australian coast and the density of sarcocystosis-positive farms increased in areas of low soil pH (intensity ratio: 0.86, 95% CI: 0.78, 0.95) and high clay content. We hypothesize that region was confounded by, and predominately acted as a proxy for, cat density. Our results have broader implications regarding the health, welfare, economic, and conservation impacts of other cat-borne parasitosis, such as toxoplasmosis.

## Introduction

Feral and domestic cats (*Felis catus*) harbor a range of infectious diseases that impact on the health and welfare of human and animal hosts (1). However, despite their importance, the majority of studies on cat-borne diseases are of limited scale, focusing on relatively small populations, and relatively small numbers of locations. Studies describing or investigating the ecology of cat-borne diseases at a landscape scale are scarce.

In agricultural areas, cats transmit infections to food animals. Food animals are systematically inspected pre- and post-slaughter for clinical conditions to control and ensure food quality and safety. When inspection findings are centrally recorded, they can provide insights into the frequency and distribution of food animal diseases across wide geographical areas, including infections of feline origin. One of these conditions, sarcocystosis, can affect meat aesthetics and quality, but does not threaten consumer health (i.e., it is not zoonotic). Sarcocystosis is caused by a protozoan parasite in the genus *Sarcocystis*. *Sarcocystis spp*. generally have a two-host predator-prey lifecycle, where the carnivorous definitive host predates on an intermediate host (2). In the intermediate host, the parasites develop into cysts (termed sarcocysts) within the skeletal musculature, that vary in size depending on the *Sarcocystis spp*. Large sarcocysts that are visible to the naked eye are described as “macroscopic,” whereas those not visible to the naked eye are described as “microscopic.”

Macroscopic sarcocystosis in sheep (*Ovis aries*) (intermediate host) is caused by two parasites, *S. gigantean*, and *S. medusiformis*. The sexual reproduction of these two parasites occurs in the digestive tract of the domestic cat, after which sporocysts are shed in the feces (3, 4). Sporocysts can survive for ~6–8 months in the environment (5, 6); they are resistant to freezing (7, 8), and killed by desiccation (5), although survival is also influenced by humidity (6). The impact of other environmental factors on sporocyst survival is not well-known. Sheep are exposed and infected by consuming contaminated pasture, water or soil (9). Parasites develop into macroscopic sarcocysts in the musculature which are detected and recorded during visual inspection of sheep carcasses at the slaughterhouse (10, 11). At slaughter, carcasses infected with sarcocystosis are trimmed, or in the case of highly infected carcasses, the whole carcass is condemned for human consumption (12). Trimmed carcasses are then boned-out to facilitate sarcocyst removal, and consequently sold as a lower quality product. Macroscopic sarcocystosis results in economic losses for both sheep farmers and meat processors (13).

In 2007, Primary Industries and Regions South Australia initiated an ongoing slaughterhouse surveillance program to monitor sarcocystosis and other health conditions of sheep farmed across South Australia. Inspection data are recorded from any farm submitting sheep directly to two major slaughterhouses within the state. This provides continuous information, spanning a large, and diverse geographical area, with substantial variation in environmental and climatic factors to investigate the ecology of cat-borne diseases.

Our objective was to analyse the slaughterhouse surveillance data to investigate: (1) the geographic distribution of sarcocystosis, and (2) identify potential location-dependent risk factors associated with the occurrence of the disease. We mapped the geographic distribution of sarcocystosis throughout South Australia and investigated potential associations with geographic and climatic factors. A better understanding of geographic and climatic factors associated with the presence of infection might support the development of targeted sarcocystosis control programs.

## Materials and Methods

### Study Population: Slaughterhouse Surveillance Data

The sarcocystosis data used in our study were collated by Primary Industries and Region South Australia over the period January 2nd 2007 to December 31st 2017. This dataset includes all sheep that were directly consigned (i.e., there were no intermediate movements between farm and slaughterhouse) for slaughter at the Thomas Foods International slaughterhouses in Murray Bridge and Lobethal, South Australia.

The slaughterhouse surveillance dataset captures prevalence estimates by meat inspectors of visible sarcocysts in dressed carcasses at the flock-level (14). In the remainder of the paper we use the term “farm” to refer to a geographical area in which sheep are reared, and “flock” as a consignment of sheep submitted for slaughter at a slaughterhouse. Using these definitions, multiple flocks can originate from a single farm throughout the 11 year study period. These data do not include information on visible sarcocysts in the offal of sheep and do not differentiate between carcasses with single or multiple sarcocysts.

**Figure 1:**
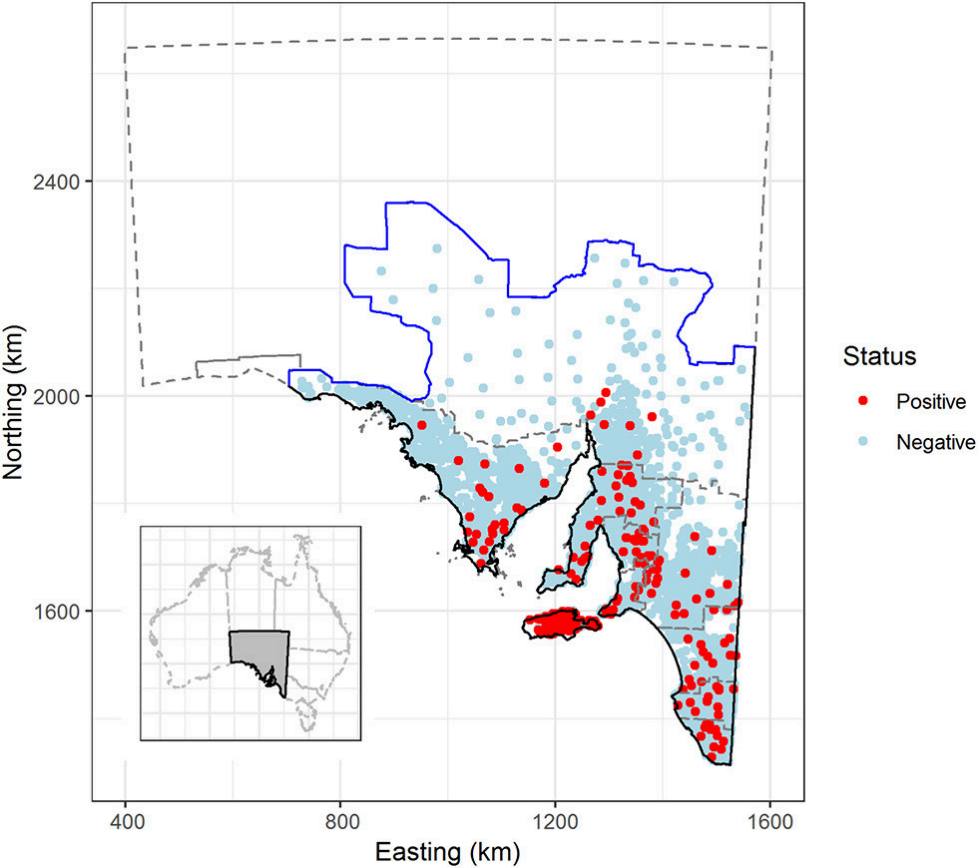
Sarcocystosis-positive and negative sheep farms, based on sheep submitted to the Thomas Foods International slaughterhouses in South Australia, 2007–2017. Solid black line defines the extent of the study region; the solid blue line represents the dog fence and outlines the northern and western boundary of the study region; the gray dashed line outlines farm identification code regions and the South Australian state border. Farm locations have been jittered by 5 km to obscure the identity of farm locations in remote areas. Insert—location of South Australia in relation to the remainder of Australia. Map projection EPSG: 3107 South Australian Lambert, GDA 94.

We only analyzed data collected for sheep that were >2 years of age. The prevalence of macroscopic sarcocysts in lambs, defined as sheep <2 years of age, is very low due to the risk of infection being cumulative and sarcocysts taking time to grow to a visible size (15). We further restricted the study population to include only those sheep farms that were located to the south of the wild dog barrier fence in South Australia (Figure 1). The dog fence runs continuously for 2,132 km (5,614 km total fence length) across the middle of the state from east to west, and was built to protect sheep flocks from dingo (*Canis lupus dingo*) predation. Relatively few sheep farms are present to the north of the dog fence as a consequence of dingo predation and the effect of excluding these farms on the inferences drawn from our analyses was reasoned to be low. All sheep included in our study were assumed to have originated from extensive grazing systems as relatively few farms in South Australia operate other systems (feedlots or similar). We are not aware of any parasite treatments for sheep or cats that could influence the disease status of sheep or farms.

### Data Formatting and Structure

The raw slaughterhouse surveillance data provided information at the flock level. Flock level data are linked back to their farm of origin by a unique farm identification code, which is given to all South Australian farms submitting sheep to the slaughterhouse. Farm identification codes are managed via a hierarchical system where they are grouped into zones, and zones grouped into regions. South Australia is divided into 10 farm identification code regions; (1) Adelaide Hills/Fleurieu Peninsula, (2) Barossa Valley/Lower North, (3) Eyre Peninsula, (4) Kangaroo Island, (5) Lower South East, (6) Mid-South East, (7) Murray Mallee, (8) Northern Pastoral, (9) Upper-South East, and (10) Yorke Peninsula/Mid-North. In the remainder of this paper we use the term “region” to refer to farm identification code region.

We used the slaughterhouse surveillance data on the total number of sheep within each submitted sheep flock and the estimated sarcocystosis prevalence for each flock, to back-calculate the number of sarcocystosis-positive sheep in each flock. A farm-level dataset was created by pooling sheep-level data to describe: (1) the number of sarcocystosis-positive sheep submitted by each farm over the 11-year study period; (2) the number of sheep submitted by each farm (irrespective of disease status) over the 11-year study period; (3) the farm-level period prevalence of sarcocystosis across the 11-year study period; and (4) the latitude and longitude of the centroid of each farm. The farm-level data (farm locations and the information associated with each location) were used to create two marked point pattern datasets using the spatstat package (16) in R version 3.5.1 (17). In this context the term “marked” refers to attribute information (e.g., the total number of sheep slaughtered by each farm over the study period) attached to each point location. Two marked point pattern datasets were created, one for the sarcocystosis-positive farms, and one for the entire farm population at risk over the 11 year study period.

### Data Analyses

#### Case Definition and Spatial Unit of Interest

Our spatial unit of interest was the centroid of a given sheep farm. We considered a farm a case/sarcocystosis positive if one or more sheep submitted to the slaughterhouse by a particular farm over the entire study period had visible sarcocystosis.

#### Geographical Distribution of Sarcocystosis Farms

The spatial distribution of a disease can be classified into two components: broad-scale trends (first-order effects) and spatial dependence/interaction (second-order effects) (18). For example, broad-scale trends/first-order effects in the distribution of sarcocystosis would be predicted to occur due to temperature or rainfall influencing the survival time of *Sarcocystis* sporocysts in the environment, and spatial dependence/second-order effects, otherwise referred to as spatial autocorrelation, could occur if the disease status of one farm influenced the disease status of surrounding farms. To assess broad-scale trends and locate areas of increased period prevalence of sarcocystosis, we created a period prevalence density surface. The numerator layer was the count of sarcocystosis-positive sheep per km^2^. This layer was created by weighting each sarcocystosis-positive farm by the total number of sarcocystosis-positive sheep that the farm had submitted for slaughter over the 11-year study period. The denominator comprised the total count of sheep per km^2^. This layer was created by weighting each farm (irrespective of disease status) by the total number of sheep that the farm had submitted for slaughter over the 11-year study period. In contrast to all other analyses, we created the sarcocystosis prevalence density map at the sheep-level to account for the large variation in the number of sheep submitted to the slaughterhouse by each farm, and the potential influence of this on within-farm estimated sarcocystosis period prevalence.

Density surfaces were created using the marked planar point pattern datasets and a kernel smoothing technique. Here we used a regular grid of 300 × 300 cells (2.9 km east-west × 3.5 km north-south) superimposed over the extent of our study area, with the standard deviation of the Gaussian kernel (that is, the bandwidth) fixed at 10 km for the positive-sheep density layer and 15 km for the population sheep density layer. Our reason for using a larger bandwidth for the sheep population at risk layer was to deal with the situation where, in areas where the density of the sheep population at risk was low, small changes in the number of sarcocystosis positive sheep led to unacceptable variation in the ratio of the two kernel estimates (19, 20). The bandwidth for the sarcocystosis positive sheep density layer was determined using the cross-validation method (21). Maps showing the number of sarcocystosis positive sheep per 100 sheep per square km based on the two sheep density layers (described above) were developed using the sparr package (22) in R. Using this same process, we then created annual period prevalence density maps for years 2007–2017 inclusive to assess the assumption of stationarity (23).

The presence of spatial dependence or interaction at the farm-level was assessed by computing Ripley's empirical K-function (24, 25) for sarcocystosis-positive and sarcocystosis-negative farms. The K-function identifies the distance over which dependence between points occurs (26) and is defined as the expected number of points that are located within a distance *h* of an arbitrary selected point location, divided by the overall density of points (24). If dependence between points was detected, sarcocystosis-positive farms would likely be surrounded by other sarcocystosis-positive farms and, for small values of distance *h*, K(*h*) would be relatively large. Conversely, if sarcocystosis-positive farms were regularly spaced, each sarcocystosis-positive farm would likely be surrounded by empty space and, for small values of distance, K(*h*) would be small. To facilitate inference, we created separate K-function plots for sarcocystosis-positive and sarcocystosis-negative farm locations. For each value of *h* we calculated the K-function difference as:

(1) $$\begin{array}{l} D\left(h\right)=K{\left(h\right)}_{positive}-K{\left(h\right)}_{negative} \end{array}$$

For a given *h*, if sarcocystosis-positive farm locations were spatially aggregated more than the sarcocystosis-negative farm locations D *h* then will appear graphically as a peak, providing an indication of the nature of dependence and the distance over which it occurred within the data (20, 26).

#### Risk Factor Analysis

We compared sarcocystosis-positive farm density (number positive farms per 100 farms per km^2^) with raster maps of each of our hypothesized explanatory variables (Table 1). Explanatory variables were all expected to potentially impact the time to desiccation of *Sarcocystis* sporocysts in the environment, and consequently sporocyst survival, by increasing or decreasing sporocyst moisture loss, or rupturing or degrading the sporocyst wall in some way (2). We included region (described above) to adjust for unaccounted for variation operating at the regional level. We did not include cat density (the definitive host of macroscopic ovine sarcocystosis) as no appropriate layer existed.

**Table 1:** Candidate explanatory variables hypothesized to influence the distribution of sarcocystosis.

**Candidate explanatory variable**	**Resolution (m)**	**Date range (years)**	**Value range**	**Source**
Average annual rainfall	5,000	1961–1990	134–1019 mm	(27)
Average count of days per annum with precipitation >1 mm	2,500	1961–1990	16–125 days	(27)
Average annual relative humidity measured at 9 a.m.	10,000	1976–2005	46–78%	(27)
Average count of frost days per annum (minimum daily temperature ≤ 0°C)	5,000	1976–2005	0–32 days	(27)
Annual average of maximum daily temperature	2,500	1961–1990	17–29°C	(27)
Annual average of daily sunshine duration	25,000	1990–2003	5–9 h	(27)
Clay content in the top 0–5 cm of soil	90		3–42%	(28)
Sand content in the top 0–5 cm of soil	90		37–95%	(28)
Soil pH	90		3.3–9	(28)
Region			1–10	PIRSA^a^

a *Primary Industries and Regions South Australia*.

We plotted sarcocystosis-positive farm density as a function of each of the hypothesized explanatory variables using the rhohat procedure (29) implemented in spatstat (30). Explanatory variables were selected for multivariable modeling, based on the presence of a non-erratic (no irregular or unusual spikes), clearly defined and well-supported association with sarcocystosis-positive farm density in the rhohat plots. We tested for collinearity amongst risk factors using variance inflation factors (VIFs) implemented within the “USDM” package (31). Risk factors with VIFs exceeding a pre-selected threshold of three (32) were excluded. The possibility of two-way interactions between non-collinear candidate explanatory variables were considered, and none were judged to be biologically plausible.

Poisson point process models fitted in spatstat are expressed in terms of the Papangelou conditional intensity function (33, 34) denoted by λ ^*(u,x)*^. When referring to Poisson point process models and the Papangelou conditional intensity function, we use the terms intensity and density interchangeably, in an attempt to make our work more interpretable for non-statistically minded readers, although we recognize that intensity is more readily used within the field of spatial statistics. We assumed that the density of sarcocystosis-positive farms was a loglinear function of parameters Φ and θ (35):

(2) $$\begin{array}{l} log\lambda \left(u,x\right)=\phi^{T}b\left(u\right)+\theta^{T}S\left(u,x\right) \end{array}$$

where ϕ^*T*^*b*(*u*) represents the broad scale (first order) trend component of the conditional intensity and θ^*T*^*S*(*u,x*) represents the spatial dependence (second order) component. To capture non-linear associations between sarcocystosis-positive farm density and each of our hypothesized explanatory variables for multivariable modeling, continuous variables were categorized based on the rhohat plot trends (described above). To model broad-scale trends, we included an offset term representing the geographic distribution of all farms that submitted sheep for slaughter throughout the study period, in addition to each of the risk factors identified previously to be associated with sarcocystosis-positive farm density using the rhohat procedure. Our model offset term was log transformed so that it was on the appropriate scale for our loglinear model. To assess the need for a spatial dependence term in our model, we created a variogram of the standardized model residuals. In a well-fitting model, the residual variogram should be essentially flat, showing no evidence of spatial correlation (36). Model selection involved manual backwards stepwise variable selection considering Akaike Information Criterion values (37).

Outputs from the point process model were estimated regression coefficients and their 95% confidence intervals (CI) for each of the parameterised explanatory variables. For those explanatory variables that varied on a continuous scale, the exponent of the regression coefficient is interpreted as the multiplicative effect of a one unit increase in the value of the explanatory variable on sarcocystosis-positive farm density. For categorical explanatory variables, the exponent of the regression coefficient is interpreted as the multiplicative effect increase or decrease in sarcocystosis-positive farm density for that level of the factor compared to the defined reference category. Residuals from our point process model were assessed using a series of diagnostic plots (lurking variable plots) to confirm goodness-of-fit and to identify outliers in the data; all plots were implemented in spatstat (38, 39).

All figures were created in the spatstat (16) and ggplot2 (40) packages in R V3.5.1 (17).

## Results

Our restricted dataset represented a total of 3,865,608 sheep, 2 years or older, submitted for slaughter at the two study slaughterhouses originating from 17,341 flocks and from 4,204 farms across 385,468 km^2^ of South Australia's land mass (Table 2, Figure 1) over the 11-year study period. Period prevalence was low at the farm-, flock- and animal-level across the 11 year study period in all regions of the state, except Kangaroo Island (Table 2). On Kangaroo Island, the period prevalence of sarcocystosis was between 14 and 66 times greater than any other mainland region depending on which level of data hierarchy was compared (farm-level Vs. flock-level Vs. animal-level) (Table 2).

**Table 2:** Regional summary statistics at farm-, flock-, and animal-level, showing number sampled, number positive to sarcocystosis and period prevalence over 2007–2017 period.

**Region**	**Total farms**	**Positive farms**	**Farm period prevalence % (95% CI)**	**Total flocks**	**Positive flocks**	**Flock period prevalence % (95% CI)**	**Total sheep**	**Positive sheep**	**Sheep period prevalence % (95% CI)**
1 - Adelaide Hills/Fleurieu Peninsula	162	10	6.2 (3.4, 11.0)	544	12	2.21 (1.14, 3.82)	90,474	420	0.46 (0.42, 0.51)
2 - Barossa Valley/Lower North	245	12	4.9 (2.8, 8.4)	815	13	1.60 (0.85, 2.71)	158,864	572	0.36 (0.33, 0.39)
3 - Eyre Peninsula	882	26	2.9 (2.0, 4.3)	3,987	27	0.68 (0.45, 0.98)	852,490	1,347	0.16 (0.15, 0.17)
4 - Kangaroo Island	310	266	85.8 (81.5, 89.3)	2,475	1,720	69.49 (67.64, 71.31)	502,559	165,244	32.9 (32.8, 33.0)
5 - Lower South East	214	12	5.6 (3.2, 9.5)	801	13	1.62 (0.87, 2.76)	209,835	394	0.19 (0.17, 0.21)
6 - Mid-South East	283	13	4.6 (2.7, 7.7)	1,111	16	1.44 (0.83, 2.33)	326,934	669	0.20 (0.19, 0.22)
7 - Murray Mallee	671	22	3.3 (2.2, 5.0)	2,480	26	1.05 (0.69, 1.53)	494,355	1,177	0.24 (0.22, 0.25)
8 - Northern Pastoral	327	7	2.1 (1.0 4.4)	1,496	10	0.67 (0.32, 1.23)	459,976	515	0.11 (0.10, 0.12)
9 - Upper-South East	347	15	4.3 (2.6, 7.0)	964	16	1.66 (0.95, 2.68)	264,417	764	0.29 (0.27, 0.31)
10 - Yorke Peninsula/Mid-North	763	30	3.9 (2.8, 5.6)	2,668	47	1.76 (1.30, 2.34)	505,704	1,578	0.31 (0.30, 0.33)
Total	4,204	413		17,341	1,900		3,865,608	172,680	

**Figure 2:**
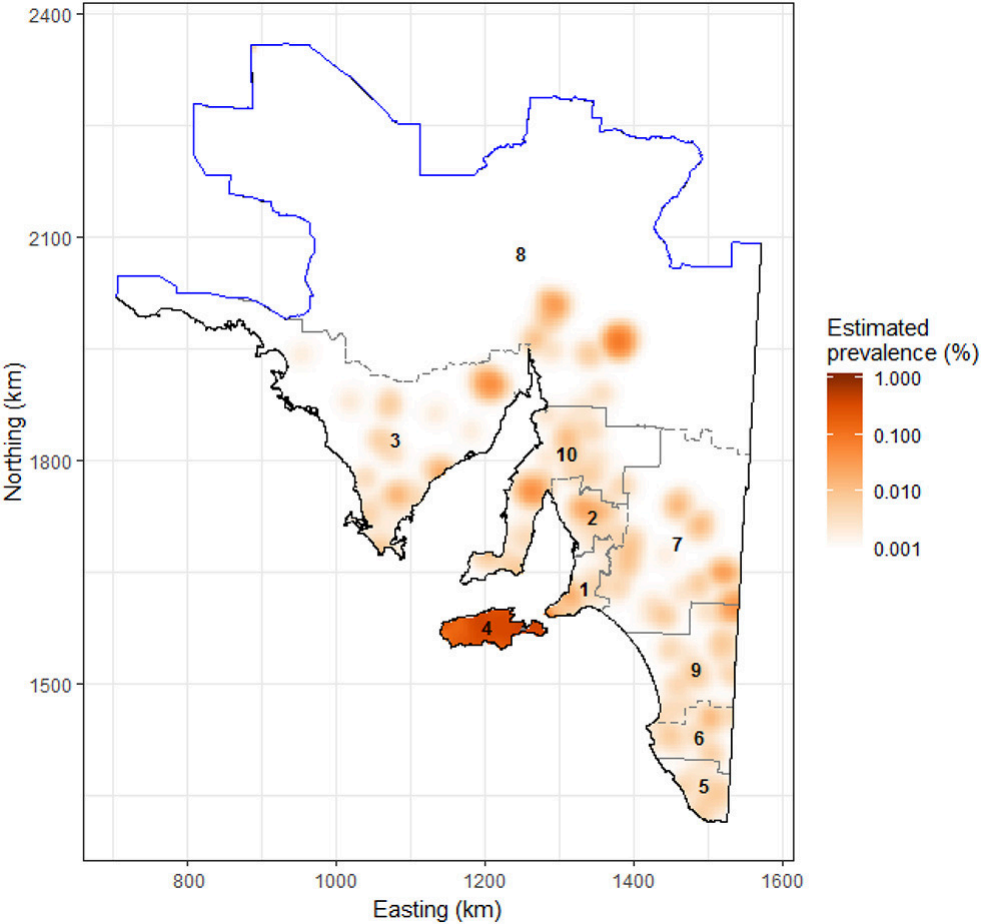
Raster image showing the estimated prevalence of sarcocystosis in sheep >2 years of age submitted to the Thomas Foods International slaughterhouses in South Australia, 2007–2017. Data were log-transformed for plotting to facilitate detection of high- and low-risk areas, as the estimated prevalence of sarcocystosis on the mainland is relatively low. The solid blue line represents the dog fence and outlines the northern and western boundary of the study region; the gray dashed line delineates the farm identification code regions/numbers as listed in Tables 2, 3. Map projection is EPSG: 3107 South Australian Lambert, GDA 94.

*Region numbers correspond to those shown in Figure 2 and Table 3*.

**Table 3:** Estimated regression coefficients and their standard errors from the reduced Poisson point process model of variables associated with sarcocystosis-positive farm density.

**Explanatory variable**	**Coefficient (SE)**	***P*-value**	**Density ratio^a^ (95% CI)**
Intercept	11.49 (0.42)		
**Soil pH**	**−0.15 (0.05)**	**0.003**	**0.86 (0.78, 0.95)^b^**
**Soil clay content (%)**			
<14.5	Reference		1.0
≥14.5 <16.5	0.24 (0.13)	0.07	1.27 (0.99, 1.63)
**≥16.5**	**0.37 (0.14)**	**0.008**	**1.45 (1.10, 1.92)**
**Region (number – name):^c^**			
1 - Adelaide Hills/Fleurieu Peninsula	Reference		1.0
2 - Barossa Valley/Lower North	0.09 (0.43)	0.83	1.09 (0.47, 2.52)
3 - Eyre Peninsula	−0.44 (0.39)	0.27	0.65 (0.30, 1.40)
**4 - Kangaroo Island**	**2.72 (0.33)**	**<0.001**	**15.25 (8.04, 28.94)**
5 - Lower South East	0.22 (0.44)	0.62	1.25 (0.52, 2.98)
6 - Mid-South East	−0.08 (0.43)	0.85	0.92 (0.40, 2.14)
7 - Murray Mallee	−0.19 (0.40)	0.65	0.84 (0.38, 1.82)
8 - Northern Pastoral	−0.85 (0.50)	0.09	0.43 (0.16, 1.14)
9 - Upper-South East	−0.07 (0.41)	0.86	0.93 (0.42, 2.08)
10 - Yorke Peninsula/Mid-North	−0.29 (0.38)	0.45	0.75 (0.35, 1.59)

*Data represent sheep more than 2 years old submitted to the Thomas Foods slaughterhouses in South Australia, 2007–2017. Bold, significant factors. SE, standard error; CI, confidence interval*.

a *Density ratio equals the exponent of the estimated regression coefficient for each explanatory variable*.

b *Interpretation: after controlling for the confounding effect of region in which a farm was located, one unit increases in soil pH decreased the density of sarcocystosis-positive farms by a factor of 0.86 (95% CI 0.78–0.95)*.

c *Region refers to farm identification code regions used by Primary Industries and Regions South Australia, and corresponds to those shown in Figure 2 and Table 2*.

By visual inspection of annual sarcocystosis period prevalence density maps, there was no obvious or dramatic shift in the spatial distribution of sarcocystosis across years 2007–2017, suggesting a largely stationary point pattern. The average sarcocystosis period prevalence density map (years 2007–2017 grouped together) identified Kangaroo Island as having a substantially increased occurrence of sarcocystosis compared to the remainder of the state (Figure 2). Using the empirical K-function, we identified spatial dependence in our dataset, consistent with clustering, up to a distance of 500 m from a given farm location.

**Figure 3:**
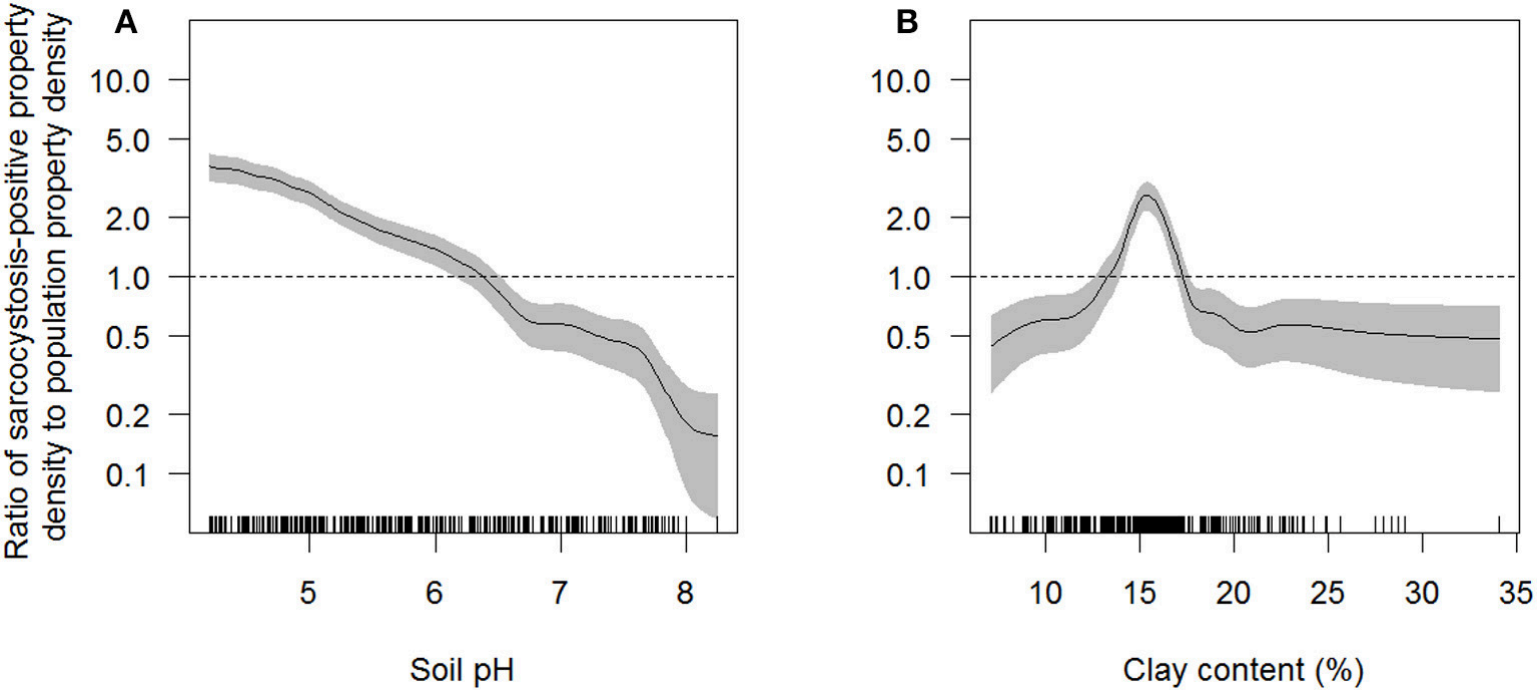
Ratio of sarcocystosis-positive farm density to population farm density as a function of soil pH **(A)** and clay content (%) in the top 0–5 cm of soil **(B)** estimated across South Australia using the rhohat procedure. The solid line shows function estimate; gray shading is pointwise 95% confidence band. The vertical dashes along the horizontal axis represent individual data points. The horizontal dashed line represents the null association (density of sarcocystosis-positive farms equals the density of all farms at risk). Interpretation: for those areas in the study area where soil pH was ~5 the intensity of sarcocystosis-positive farms was ~2 times that of all farms at risk. Data represent 4,204 sheep farms that submitted sheep for slaughter during the period 2007–2017.

**Figure 4:**
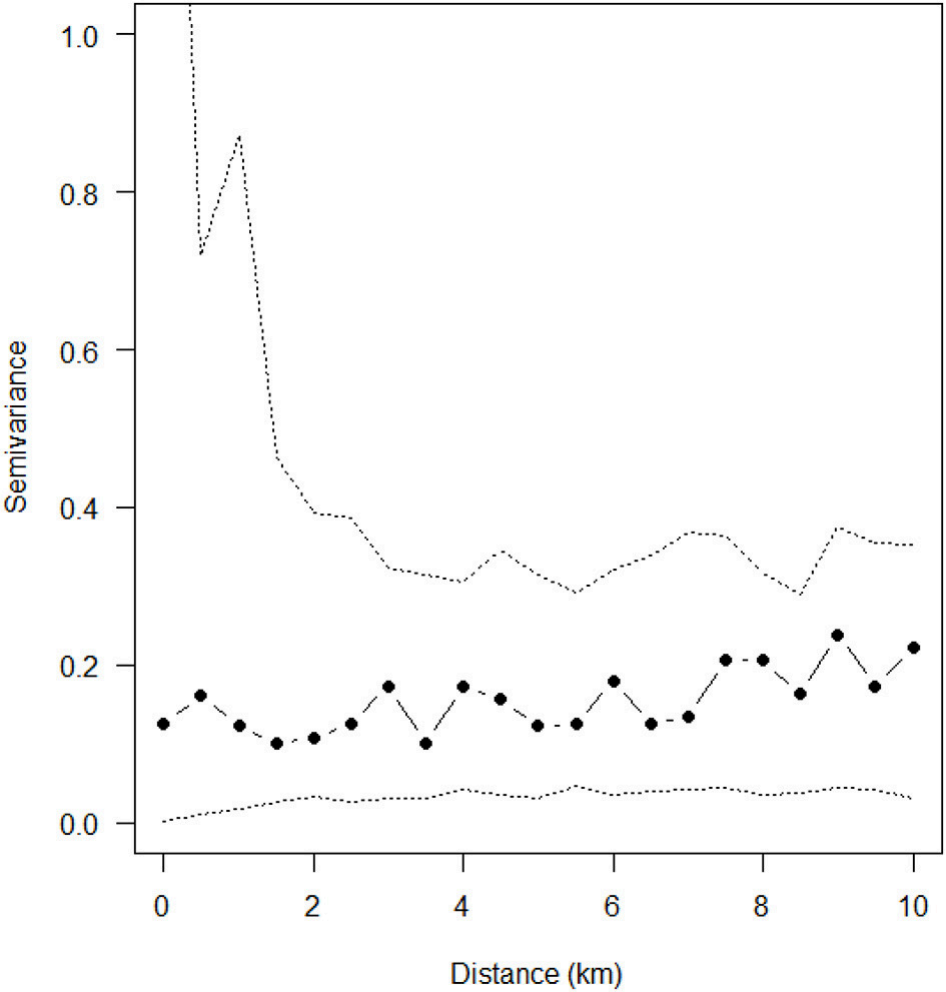
Empirical variogram fitted to the posterior mean of the standardized residuals from reduced Poisson point process model explaining sarcocystosis-positive farm density. Dashed lines show the pointwise 95% posterior intervals constructed from 999 simulated realizations of the fitted spatial model.

Soil pH, clay content in the top 0–5 cm of soil, and region were the only explanatory variables that significantly influenced (*p* < 0.05) sarcocystosis-positive farm density in the reduced Poisson point process model (Table 1 and Table S1, Figure 3). One unit increases in soil pH decreased the density of sarcocystosis-positive forms by a factor of 0.86 (95% CI 0.78–0.95). In contrast, sarcocystosis-positive farm density increased by a factor of 1.45 (95% CI 1.10–1.92) where soil clay content was ≥16.5% in the top 0–5 cm of soil relative to areas where clay content was <14.5% in the top 0–5 cm of soil. The density of sarcocystosis-positive farms on Kangaroo Island was 15.25 (95% CI 8.04–28.94) times greater than the density of sarcocystosis-positive farms located in the Adelaide Hills/ Fleurieu Peninsula. The empirical variogram lies within 95% posterior limits throughout the plotted region, demonstrating that the fitted model adequately accounted-for the second-order structure in the data (Figure 4). Whilst other explanatory variables appeared to be associated with sarcocystosis risk in the rhohat plots (Figure S1), they did not increase the explanatory power of our model. For example, soil sand content appeared to influence sarcocystosis-positive farm density (Figure S1), although spikes in the plot corresponded with soil sand content values found across the majority of Kangaroo Island.

## Discussion

Our analyses show a marked heterogeneous distribution of macroscopic ovine sarcocystosis across South Australia with a clear hotspot on Kangaroo Island. Kangaroo Island had a modeled density of sarcocystosis-positive farms ~15 times higher than the Adelaide Hills/Fleurieu Peninsula region and 12 times higher than any other region. In addition to a regional difference, the occurrence of sarcocystosis was decreased by alkaline soils and increased by soil clay content. A one unit increase in soil pH corresponded to a 14% reduction in the density of sarcocystosis-positive farms, and sarcocystosis-positive farm density increased by ~45% in soils with ≥ 16.5% soil clay content in the top 0–5 cm of soil relative to soils with <14.5% soil clay content.

Kangaroo Island is situated ~14 km off the South Australian coast line, with the Adelaide Hills/Fleurieu Peninsula region being the closest mainland region. Both Kangaroo Island and the Adelaide Hills/Fleurieu Peninsula have similar Mediterranean climates (41, 42), land uses (predominately agriculture) and vegetation communities (41, 43). One major difference between these two regions is the abundance of feral cats. We recently identified an ~11-fold greater relative abundance of feral cats on Kangaroo Island compared with the Fleurieu Peninsula (44). Cats are the definitive host of *S. gigantea* and *S. medusiformis* (3, 4), the parasites responsible for macroscopic sarcocystosis in sheep. We therefore suspect that region predominately acts as a proxy for cat density within the Kangaroo Island and Adelaide Hills/Fleurieu Peninsula regions, and likely across South Australia. We expect that active and consistent cat management on Kangaroo Island would produce long-term reductions in macroscopic sarcocystosis in the island's sheep, with cat eradication expected to result in the complete eradication of sarcocystosis by means of breaking the parasite's life cycle. Whilst other methods of reducing sarcocystosis burden on Kangaroo Island are potentially possible, such as collecting, burying or burning sheep carcasses and offal on farms, or treating all cats with anti-parasitic drugs, these methods are not practical or feasible, particularly at large geographic scales, and where large populations of feral cats exist.

We believe our study is the first to describe the impact of soil pH and clay content on sarcocystosis in sheep, and first to provide evidence from the field suggesting that soil pH and clay content may influence the survival of *Sarcocystis spp*. sporocysts in the environment. Similar relationships have however been reported for parasites closely related to *Sarcocystis spp*. For example, there is evidence that increased soil pH decreases the probability of detecting oocysts of *Cryptosporidium spp*. in the soil (45), although another study found no evidence for a relationship between soil pH and *Cryptosporidium spp*. oocyst viability (46). Similarly, soil clay content has previously been suggested, to influence the survival of *Toxoplasma gondii* oocyst in the environment (47), and *Cryptosporidium parvum* oocysts are known to associate with clay particles over time (48) due to their higher cation exchange capacity (49), although experimental studies have suggested that *Cryptosporidium parvum* oocysts have reduced survival in silt clay loam, compared to silt loam and loamy sand (50). If the viability of *Sarcocystis spp*. sporocysts does decrease with increasing soil pH, as suggested by our study, the spreading of agricultural lime across pastures may be used to mitigate macroscopic sarcocystosis in sheep. Agricultural lime is commonly spread across pastures to increase soil pH and reverse soil acidification (51) and could additionally be used to decrease the survival of *Sarcocystis spp*. sporocysts in the soil. The re-application of lime may however be necessary for long-term reductions in disease occurrence, deeming it un-feasible. Furthermore, the effectiveness of agricultural lime to reduce the survival of *Sarcocystis spp*. sporocysts at a large scale remains to be tested.

We found that the average count of frost days per annum (minimum daily temperature ≤ 0°C) did not influence the density of sarcocystosis-positive farms, consistent with previous findings that *Sarcocystis spp*. sporocysts are resistant to freezing (7, 8). *Sarcocystis spp*. sporocysts have previously been suggested to be killed by desiccation (5, 6) and candidate explanatory variables were selected based on predictions that they may influence the time to desiccation of sporocysts. However, the availability and resolution of candidate explanatory variable data may have negatively impacted on our ability to detect interacting factors and to more precisely tease out the influence of two competing variables in our analysis, for example the influence of rainfall or temperature Vs. cat density on sarcocystosis risk. We suspect that an adequate cat density data layer would be particularly beneficial in future analyses to help tease out possible variable interactions. Whilst soil sand content appeared to influence sarcocystosis risk in the rhohat plots, spikes in the plot corresponded with soil sand content values found across the majority of Kangaroo Island, explaining why this variable was not included in our reduced model.

We did not include a spatial dependence term in our model, due to the empirical variogram of the standardized residuals from the fitted model demonstrating no evidence of spatial clustering without the inclusion of this term, but we did identify spatial dependence in our data out to a distance of ~500 m using Ripley's empirical K-function. Spatial dependence is most commonly observed for infectious diseases, and represents the influence of an infected property/animal on the disease status of surrounding properties/animals. Sarcocystosis is however a non-infectious disease, and hence spatial dependence in our data suggests that cats consume *Sarcocystis* sarcocysts in infected sheep on one property and subsequently shed infective sporocysts into the environment on adjacent properties out to a distance of ~500 m. Whilst cat home ranges throughout our study region are known to have a greater radius than 500 m (52), this distance may be broadly representative of the radius of the average cats' core home range area, particularly on Kangaroo Island where cat density is high (46) and the majority of sarcocystosis-positive farms cluster.

Identifying the possible influence of soil pH and clay content on sarcocystosis is one example of where landscape scale studies can provide insights into little known aspects of the ecology of cat-borne diseases. One obvious limitation to conducting landscape scale studies is the availability of suitable landscape data of adequate scale and resolution. For example, we could not access an appropriate cat density or ultraviolet radiation layer that provided sufficient scale, resolution, quality, and variability across our study area to be of use in risk factor analysis; despite both variables being hypothesized to be important in explaining the distribution of sarcocystosis. In the absence of landscape scale data, proxies can provide appropriate insights if a strong association between the proxy and the outcome of interest has been demonstrated. The *Sarcocystis spp*. responsible for the development of macroscopic sarcocystosis in sheep are particularly closely related to *Toxoplasma gondii*, and both share similar biology and lifecycles. Consequently, we would predict that macroscopic sarcocystosis in sheep could potentially be utilized as a proxy of *T. gondii* infection in sheep, and that our findings are of relevance to other protozoal parasites other than *Sarcocystis spp*., although this association remains to be tested.

In our study, the influence of region on macroscopic sarcocystosis in sheep indicates unequal economic impacts throughout the sheep industry for this disease. We are aware of one regional slaughterhouse in South Australia that no longer processes Kangaroo Island sheep >2 years of age due to its high occurrence of macroscopic sarcocystosis. Whilst we have highlighted two potential methods of reducing sarcocystosis burden in sheep, it is likely that cat management is currently the most feasible and sustainable.

## Author Contributions

All authors contributed to study design, data analysis and/or interpretation, and manuscript writing. PT, MS, and CC additionally contributed to data collection and formatting.

### Conflict of Interest Statement

The authors declare that the research was conducted in the absence of any commercial or financial relationships that could be construed as a potential conflict of interest.
