# Supplementary material for: Spatial Analysis of a Cat-Borne Disease Reveals That Soil pH and Clay Content Are Risk Factors for Sarcocystosis in Sheep
Source: Front Vet Sci. 2019 Apr 24;6:127. doi: 10.3389/fvets.2019.00127 (PMC6491573; doi:10.3389/fvets.2019.00127)
Supplement: Supplementary file 1 [file Data_Sheet_1.docx]

**Title: Spatial mapping of a cat-borne disease reveals that soil pH and clay content is a risk factor for sarcocystosis in sheep**

**Authors:** Patrick L. Taggart^1*^, Mark A. Stevenson^2^, Simon M. Firestone^2^, Milton M. McAllister^1^, Charles G. B. Caraguel^1^

**Affiliations:**

^1^ School of Animal and Veterinary Sciences, The University of Adelaide, Roseworthy, South Australia 5371, Australia

^2^ Asia-Pacific Centre for Animal Health, Melbourne Veterinary School, Faculty of Veterinary and Agricultural Sciences, The University of Melbourne, Parkville, Victoria 3010, Australia

**Supplementary material**

**Table S1:** Model selection process showing backwards step-wise variable selection considering AIC values. Bold model coefficients and p-values indicates statistical significance.

|  | **Model 1** | | **Model 2** | | **Model 3** | | **Model 4** | |
| --- | --- | --- | --- | --- | --- | --- | --- | --- |
| **Variable** | Coefficient (SE) | P-value | Coefficient (SE) | P-value | Coefficient (SE) | P-value | Coefficient (SE) | P-value |
| *Intercept* | 11.52 (0.54) |  | 11.66 (0.46) |  | 11.63 (0.46) |  | 11.49 (0.43) |  |
| *Soil pH* | **-0.15 (0.05)** | **0.005** | **-0.16 (0.05)** | **0.004** | **-0.15 (0.05)** | **0.004** | **-0.15 (0.05)** | **0.003** |
| *Average annual rainfall (mm)* |  |  |  |  |  |  |  |  |
| < 480 | Reference |  |  |  |  |  |  |  |
| ≥ 480 | 0.12 (0.23) | 0.61 |  |  |  |  |  |  |
| *Average annual frost days* |  |  |  |  |  |  |  |  |
| < 1 | Reference |  | Reference |  |  |  |  |  |
| ≥ 1 | -0.11 (0.20) | 0.59 | -0.10 (0.20) | 0.62 |  |  |  |  |
| *Soil clay content (%)* |  |  |  |  |  |  |  |  |
| < 14.5 | Reference |  | Reference |  | Reference |  | Reference |  |
| ≥ 14.5 < 16.5 | 0.20 (0.14) | 0.14 | 0.20 (0.14) | 0.14 | 0.20 (0.14) | 0.14 | 0.24 (0.13) | 0.07 |
| ≥ 16.5 | 0.29 (0.16) | 0.07 | 0.29 (0.16) | 0.06 | 0.30 (0.16) | 0.06 | **0.37 (0.14)** | **0.008** |
| *Soil sand content (%)* |  |  |  |  |  |  |  |  |
| < 73 | Reference |  | Reference |  | Reference |  |  |  |
| ≥ 73 < 77.2 | -0.15 (0.16) | 0.35 | -0.15 (0.16) | 0.32 | -0.15 (0.16) | 0.35 |  |  |
| ≥ 77.2 | -0.20 (0.17) | 0.24 | -0.20 (0.17) | 0.22 | -0.20 (0.17) | 0.23 |  |  |
| *Pic region* |  |  |  |  |  |  |  |  |
| Adelaide Hills/Fleurieu Peninsula | Reference |  | Reference |  | Reference |  | Reference |  |
| Barossa Valley/Lower North | 0.17 (0.45) | 0.71 | 0.11 (0.43) | 0.80 | 0.07 (0.43) | 0.87 | 0.09 (0.43) | 0.84 |
| Eyre Peninsula | -0.30 (0.43) | 0.48 | -0.38 (0.40) | 0.33 | -0.39 (0.40) | 0.32 | -0.44 (0.39) | 0.27 |
| Kangaroo Island | **2.76 (0.33)** | **<0.001** | **2.77 (0.33)** | **<0.001** | **2.78 (0.33)** | **<0.001** | **2.72 (0.33)** | **<0.001** |
| Lower South East | 0.26 (0.45) | 0.55 | 0.27 (0.45) | 0.55 | 0.24 (0.44) | 0.59 | 0.22 (0.44) | 0.62 |
| Mid-South East | 0.00 (0.45) | 1.00 | 0.00 (0.45) | 1.00 | -0.06 (0.43) | 0.90 | -0.08 (0.43) | 0.85 |
| Murray Mallee | 0.06 (0.47) | 0.90 | -0.05 (0.42) | 0.91 | -0.11 (0.40) | 0.78 | -0.18 (0.40) | 0.65 |
| Northern Pastoral | -0.70 (0.56) | 0.21 | -0.81 (0.52) | 0.12 | -0.89 (0.50) | 0.77 | -0.85 (0.50) | 0.88 |
| Upper-South East | 0.10 (0.44) | 0.83 | 0.06 (0.44) | 0.90 | -0.02 (0.41) | 0.97 | -0.07 (0.41) | 0.86 |
| Yorke Peninsula/Mid-North | -0.14 (0.43) | 0.74 | -0.23 (0.40) | 0.56 | -0.28 (0.38) | 0.46 | -0.29 (0.38) | 0.45 |
| *AIC* | 4034.8 | | 4033.1 | | 4031.3 | | 4028.8 | |


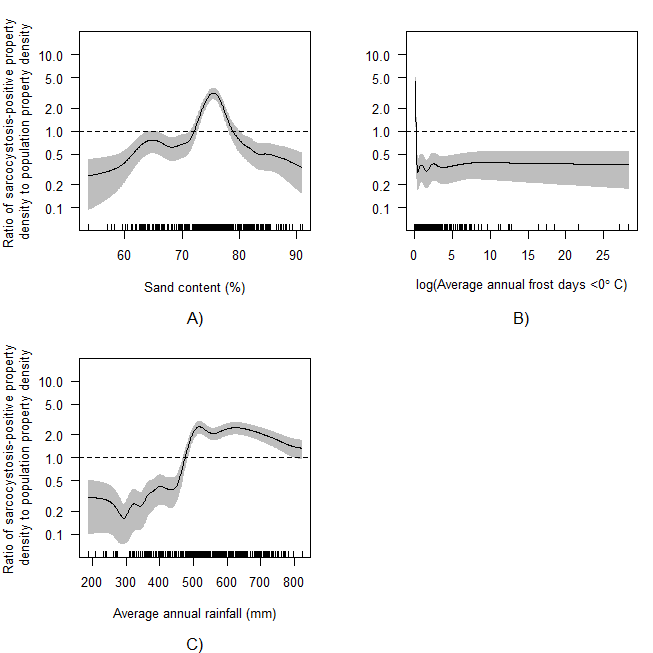


Figure S1: Rhohat plots for remaining candidate explanatory variables (excluding region) included in saturated model. Note that all other candidate explanatory variables described in methods were excluded prior to model construction due to coarseness of data or collinearity. Plots show ratio of sarcocystosis-positive farm density to population farm density as a function of soil sand content (A), average annual frost days <0 ^o^C (B), and average annual rainfall (C), estimated across South Australia using the rhohat procedure. The solid line shows function estimate; grey shading is pointwise 95% confidence band. Vertical dashes along the x-axis represent individual data points, and provide an indication of where the function is based on raw data or purely predictive. Horizontal dashed line represents null association (intensity of sarcocystosis-positive farms equals the intensity of all farms at risk). Interpretation: for those areas in the study area where average annual rainfall was ~500mm the intensity of sarcocystosis-positive farms was ~2 times that of all farms at risk. Data represent 4,204 sheep farms during the period 2007-2017.
